# Disease Characteristics and Management of Intrabiliary Colorectal Liver Metastasis: An Updated Systematic Review

**DOI:** 10.3390/jpm16080436

**Published:** 2026-08-20

**Authors:** Panagiotis Dorovinis, Konstantinos Kossenas, Anna Paspala, Dimitrios Papaconstantinou, Myrto D. Keramida, Dimitrios K. Vlachos, Dionysios Prevezanos, Stylianos Kykalos, Nikolaos Machairas, Georgios C. Sotiropoulos

**Affiliations:** 1 Department of Liver Transplant and Hepatobiliary Surgery, National and Kapodistrian University of Athens, 11527 Athens, Greece; pdorovinis@gmail.com (P.D.); anna9paspala@gmail.com (A.P.); dvlachos10@gmail.com (D.K.V.); geosotirop@med.uoa.gr (G.C.S.); 2 2nd Department of Propaedeutic Surgery, National and Kapodistrian University of Athens, 11527 Athens, Greece; kossenaswork@gmail.com (K.K.); keramidamyrto@gmail.com (M.D.K.); kykalos@med.uoa.gr (S.K.); nmachair@med.uoa.gr (N.M.); 3 3rd Department of Surgery, National and Kapodistrian University of Athens, 12462 Athens, Greece; dimpapa7@gmail.com

**Keywords:** biliary, colorectal, liver metastasis, hepatectomy, morbidity, mortality

## Abstract

**Background/Objectives**: Liver metastasis develops in approximately 50% of patients with colorectal cancer. Invasion of the biliary tract from colorectal liver metastasis (CRLM) is rarely reported. Preoperative diagnosis remains elusive, prohibiting optimal surgical management. The objective of this systematic review was to summarize the clinical, radiological, pathological, and treatment characteristics of ibCRLM and describe the reported outcomes. **Methods**: A systematic literature search of the Medline, Embase, Web of Science, CENTRAL, and CINAHL databases was undertaken for studies reporting clinical outcomes of patients with ibCRLM, up to May 2026. An individual patient data analysis approach was utilized. **Results**: Thirty-eight case reports and 10 case-series, incorporating 228 patients with biliary involvement from CRLM, were identified. Mean age was 62.4 ± 10.9 years, with a male-to-female ratio of 3.2:1. The majority of metastatic lesions were metachronous in 71.1% and solitary in 59.1% of patients. Surgical treatment was implemented in 89.2% of patients. Major hepatectomy was the most common procedure, being performed in 46.2% of patients, followed by minor hepatectomy in 38.7% and pancreatoduodenectomy in 4.3%. After a median follow-up of 52.5 months (range 2–164 months), the survival rate was 60.5%. Non-survivors were found to have significantly more synchronous CRLM (50% versus 0, *p* = 0.008), while a single patient did not receive any curative-intent treatment and died 20 days following CRLM presentation. **Conclusions**: IbCRLM is a distinct clinicopathological presentation of CRLM with characteristic radiological and pathological features. The available evidence suggests that selected patients may achieve favorable long-term outcomes following complete surgical resection, although these findings should be interpreted with caution given the limitations of the available literature.

## 1. Introduction

Colorectal cancer (CRC) is a common type of malignancy worldwide, while it accounts for 151.030 newly diagnosed cases and 52.580 cancer related deaths annually in the United States [1]. Colorectal liver metastasis (CRLM) will develop at some point over the course of the disease, in up to half of the patients [2]. Advances in surgical techniques constitute resection as the treatment of choice for resectable CRLMs. In the context of multidisciplinary management, 5-year overall survival rates exceed 50% [3].

Biliary invasion from CRLM has rarely been reported as a distinct pathological entity of intrahepatic growth. Jaundice and biliary dilation are more commonly attributed to compression or invasion of the biliary tract from the metastatic tumor [4] with radiological findings mimicking those of intrahepatic cholangiocarcinoma (iCCA) with intraductal growth. However, recent advances in magnetic resonance (MR) and computed tomography (CT) imaging have added value to the preoperative diagnosis of these lesions [5], which demonstrate a favorable biological behavior and prognosis, when successfully treated with radical surgical resection [6].

**Figure 1:**
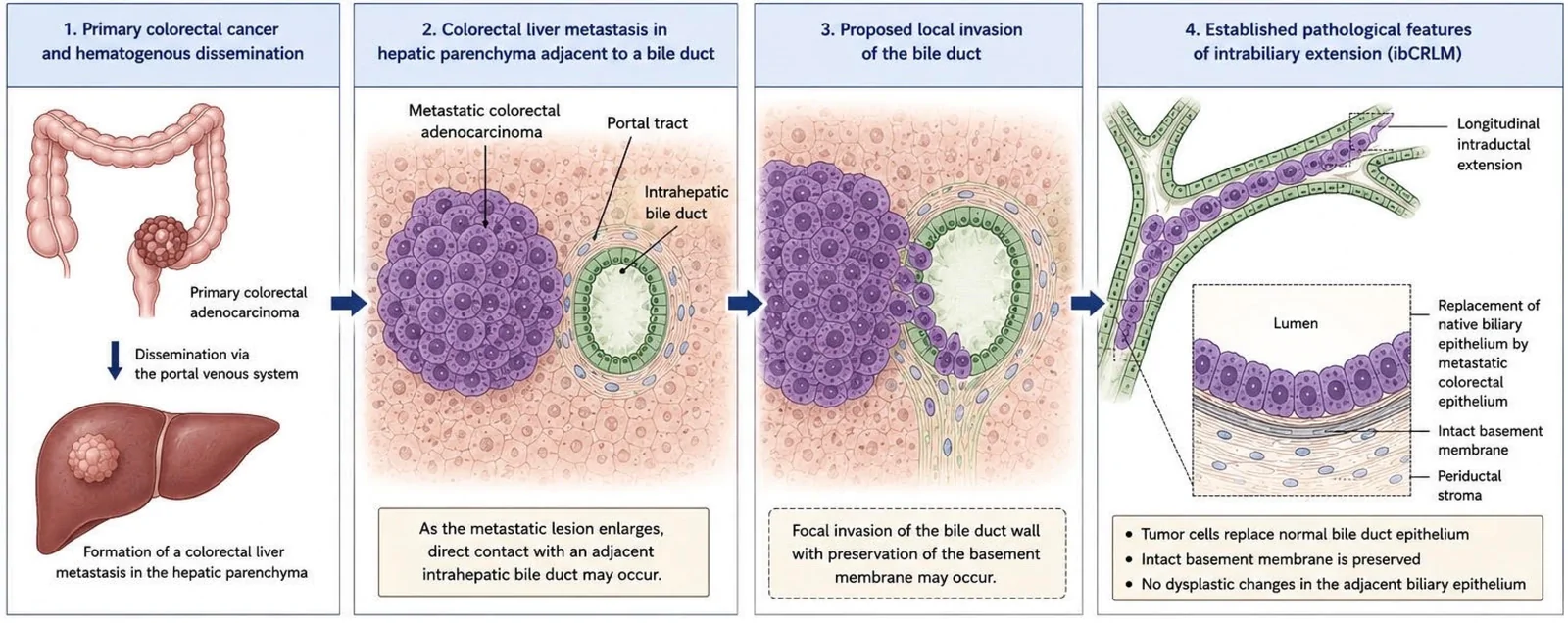
Proposed mechanism of intrabiliary extension of colorectal liver metastases. Following hematogenous dissemination to the liver, a colorectal liver metastasis develops within the hepatic parenchyma. In selected cases, tumor cells invade an adjacent bile duct, replace the native biliary epithelium, and extend longitudinally along the biliary tree. This Figure illustrates a proposed model of intrabiliary tumor extension based on the available pathological evidence.

Moreover, the pathological distinction of this disease from primary cholangiocarcinoma can also be challenging. The difficulty of distinguishing intrabiliary CRLM (ibCRLM) from iCCA using the conventional hematoxylin-eosin stain has been overcome by the implementation of immunostaining using antikeratin antibodies [7]. Histopathological studies have demonstrated that tumor cells may replace the native biliary epithelium while preserving the basement membrane, representing a characteristic pathological feature of ibCRLM. However, the biological mechanisms underlying the development of intrabiliary extension remain incompletely understood [8]. Conflicting data emerge about the prognosis of ibCRLMs. Although some studies associate intrabiliary spread with a less invasive nature of the disease [9], others emphasize the association of a higher risk of recurrence of the disease [10]. Definite preoperative diagnosis of ibCRLMs is of cardinal importance to determine the appropriate treatment approach for this specific clinical entity. Despite the accurate preoperative diagnosis using the available radiological and pathological findings, multidisciplinary team (MDT) discussion can help determine the proper surgical management of these patients [Figure 1].

The objective of our systematic review is to provide further insight into this rare clinical entity regarding preoperative diagnoses and radiological and pathological features, define the most appropriate surgical approach, and evaluate its outcomes.

## 2. Materials and Methods

### 2.1. Eligibility Criteria

The eligibility criteria were based on the PICO format (population, intervention, control, and outcomes). This systematic review included patients with histologically and/or radiologically confirmed ibCRLM (Population) treated surgically or non-surgically (Intervention). Considering the descriptive nature of the available literature, studies without a comparator group were also deemed eligible (Comparison). Eligible outcomes were clinicopathological characteristics, radiological and pathological findings, surgical management and survival outcomes (Outcomes). All primary study literature, including case reports, case series, retrospective and prospective observational studies, were eligible. Exclusion criteria were studies of non-colorectal liver metastases, lesions without confirmed intrabiliary metastasis, duplicate cohorts, reviews, editorials, letters and conference abstracts with no extractable data.

### 2.2. Search Strategy and Study Selection

A systematic literature search was performed in Medline/PubMed, Embase, Web of Science, CENTRAL and CINAHL from the date of database inception to May 2026. Search terms consisted of combinations of “intrabiliary”, “biliary”, “bile duct”, “biliary tree”, and “colorectal liver metastases” using Boolean operators (AND/OR) as appropriate for each database (Supplementary Material S1). Reference lists of eligible studies were also manually screened for potentially relevant articles. Three reviewers (PD, KK and DP) independently performed study selection. We first screened titles and abstracts for eligibility and then assessed the full text of potentially relevant studies. Discrepancies in study selection were resolved through discussion and adjudication by a third reviewer (MDK).

This systematic review was prospectively registered in the International Prospective Register of Systematic Reviews (PROSPERO; registration number CRD420261407799) and was conducted in accordance with the Preferred Reporting Items for Systematic Reviews and Meta-Analyses (PRISMA 2020) guidelines (Supplementary Material S1).

### 2.3. Data of Interest and Definitions

Three reviewers (PD, KK and DP) independently extracted individual patient-level data using standardized predefined extraction forms. Data were extracted directly from the text, Tables, Figures, and Supplementary Materials of the included studies whenever available. Discrepancies were resolved through discussion and adjudication by a fourth reviewer (MDK). Variables that were not reported in the original publication were treated as missing and excluded from analyses of that specific variable. Consequently, denominators vary throughout the manuscript according to data availability. Variables extracted were patient demographics, study characteristics, timing of metastatic presentation (synchronous or metachronous), number and size of metastatic lesions, type of biliary invasion, surgical management, resection status, follow-up duration and survival outcomes. Potential overlap between publications was assessed during data extraction by comparing the reporting institution, study period, patient demographics, tumor characteristics, and treatment details. When duplicate reporting was suspected, the publication containing the most comprehensive patient-level data was retained, and duplicate cases were excluded from the final dataset.

Data extraction was guided by predefined criteria and definitions to minimize heterogeneity in reporting. Major hepatectomy was defined as resection of four or more liver segments, while minor hepatectomy was defined as all other hepatic resections. The interval of presentation was defined as the time between treatment of the primary colorectal malignancy and the diagnosis of metachronous liver metastases. For the purpose of the present review, biliary invasion was classified according to predefined standardized criteria applied during data extraction. Macroscopic biliary invasion was defined as involvement of the common bile duct, common hepatic duct, or right/left hepatic ducts, as demonstrated clinically, radiologically, or intraoperatively. Cases without overt biliary involvement, including isolated peripheral biliary dilatation or histopathological evidence of ductal spread only, were classified as microscopic biliary invasion. Primary colorectal tumors were staged according to the American Joint Committee on Cancer (AJCC) 8th edition staging system. R0 resection was defined as the absence of tumor cells within 1 mm of the surgical margin.

### 2.4. Quality Assessment

The methodological quality of the included studies was evaluated using the Joanna Briggs Institute (JBI) Critical Appraisal Checklists for Case Reports and Case Series, respectively. Two reviewers independently assessed the quality of studies, and disagreements were resolved by consensus.

### 2.5. Statistical Analysis

An approach using individual patient data (IPD) analysis was employed. Patient-level data were extracted from the text, Tables, or Supplementary Materials of the included studies, if possible. For studies that provided Kaplan–Meier survival curves but did not provide individual survival data, survival data were reconstructed using WebPlotDigitizer softwarev5 (https://automeris.io/WebPlotDigitizer URL accessed 20 May 2026).

Baseline clinicopathological characteristics were summarized with descriptive statistics. Categorical variables were compared using Fisher’s exact test and continuous variables were compared with the Mann–Whitney U test. Overall survival was estimated using the Kaplan–Meier method based on the survival data reported in the original publications. Patients alive at the last reported follow-up were treated as censored observations, whereas patients without extractable survival information were excluded from survival analyses. Because the included studies did not uniformly define the starting point for survival assessment, survival analyses were performed according to the definitions reported in the individual publications. Follow-up duration was calculated from the individual patient data reported in the included studies and is presented descriptively as the median (range). Comparisons between survival curves were performed using the log-rank test. Statistical analysis was conducted using IBM SPSS Statistics version 26 (SPSS Inc., Chicago, IL, USA). *p* < 0.05 was considered statistically significant.

### 2.6. Justification of Methodological Approach

A quantitative meta-analysis was not performed due to significant clinical and methodological heterogeneity among the included studies, including variations in study design, definitions of biliary invasion, operative strategies, outcome reporting, and predominance of retrospective case reports and small case series.

## 3. Results

### 3.1. Included Studies

**Figure 2:**
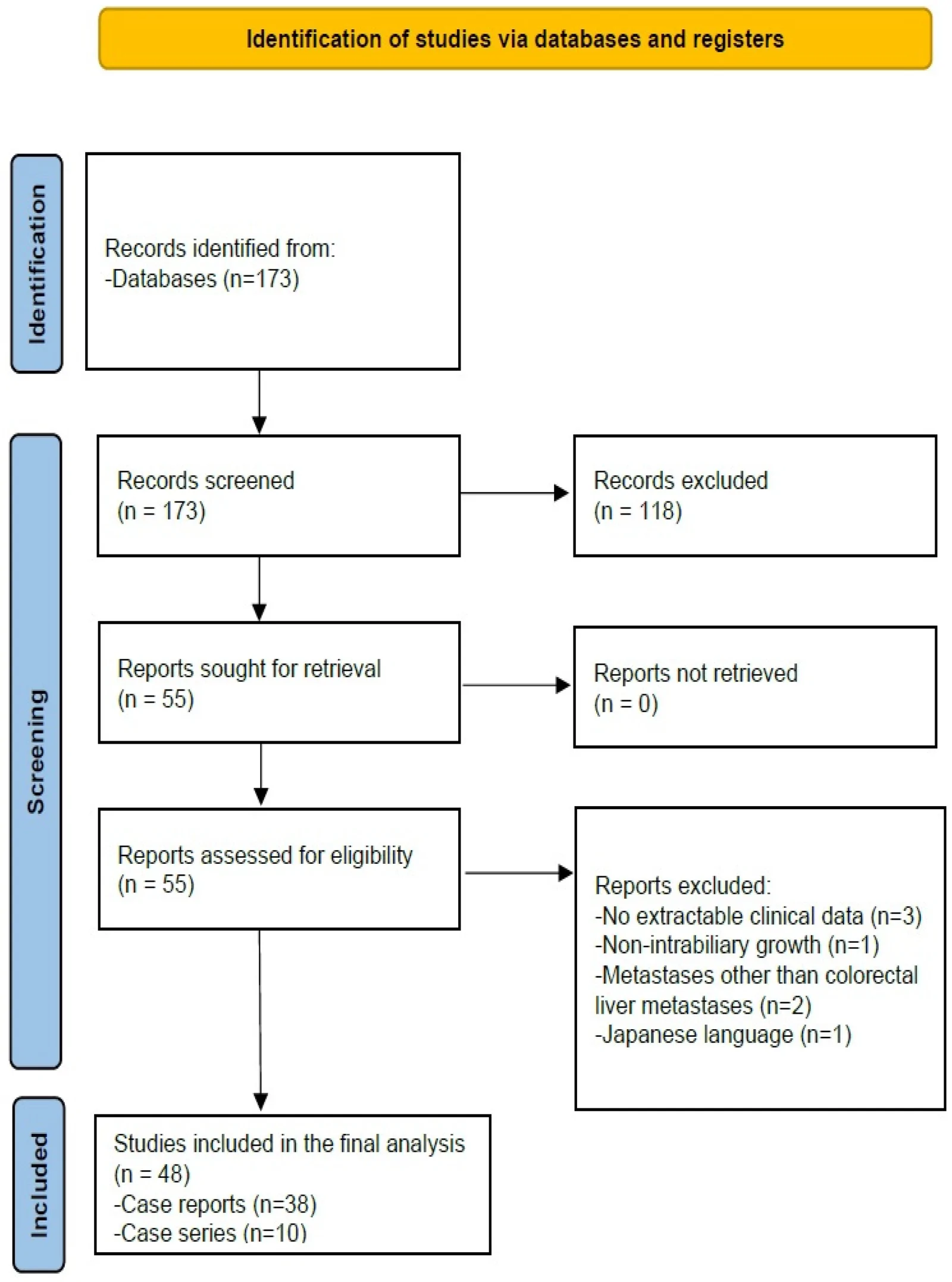
Prisma flowchart of study selection.

Our database search generated 173 studies after duplicate removal. One hundred and eighteen studies were excluded due to irrelevance. From the remaining 55 studies, three contained non-extractable data, and one failed to demonstrate intrabiliary growth, while two others contained liver metastases other than colorectal. Lastly, one study written in Japanese was excluded from our review (Figure 2).

The remaining 48 studies [4,5,6,8,9,11,12,13,14,15,16,17,18,19,20,21,22,23,24,25,26,27,28,29,30,31,32,33,34,35,36,37,38,39,40,41,42,43,44,45,46,47,48,49,50,51,52,53] consisted of 38 case reports [4,11,12,13,14,15,16,18,19,20,21,22,23,24,25,26,27,28,29,30,31,32,33,34,35,36,37,38,41,42,43,44,47,48,49,50,51,53] and 10 case series [5,6,8,9,17,39,40,45,46,52], containing 228 patients in total. Table 1 depicts the baseline characteristics of patients and their metastatic lesions. The majority of involved lesions were solitary (59.1%) with a metachronous presentation (71.1%), emerging after a median of 30 (range 4–180) months from the diagnosis of the primary colorectal tumor. Regarding the colorectal primaries, in most cases the neoplasms originated from the colon (74.2%) and were Stage II or III (28.3% and 45.3%, respectively). Amongst the evaluated patients with available data regarding ibCRLM, subcategorization as microscopic or macroscopic was possible for 205 patients, of which 120 (58.5) patients exhibited microscopic biliary spread and 85 (41.5) macroscopic. The average size of metastatic lesions was 3.2 ± 1.9 cm.

#### 3.1.1. Main Outcomes

Surgery with curative intent was performed in the majority of involved cases (89.2%), with major hepatectomy being the most commonly employed procedure (46.2%), followed by minor hepatectomy (38.7%) and pancreaticoduodenectomy (4.3%). Concurrent biliary reconstruction was necessary in 27 patients (34.2%), while palliative stenting or percutaneous transhepatic biliary drainage (PTCD) was pursued in nine patients. In a single patient not fit for surgery, endoscopic removal of a common bile duct lesion was selected for palliative purposes. Among patients with available margin data, R0 resection was achieved in 54/120 patients (45%) (Table 2).

#### 3.1.2. Macroscopic Versus Microscopic Biliary Invasion

Subanalysis of available data based on the type of biliary invasion revealed a statistically significant difference in terms of tumor presentation between the evaluated subgroups (Table 3). Specifically, ibCRLM lesions demonstrating microscopic biliary invasion were significantly more likely to be synchronously presenting and multifocal (36.4% and 54.5%, respectively) when compared to their macroscopic counterparts (7.5% and 27.7%, respectively). Macroscopic lesions were slightly larger than microscopic lesions in the available dataset (4.0 vs. 3.7 cm), although the clinical significance of this difference remains uncertain. Minor hepatectomies were significantly more commonly performed for microscopically invasive tumors, while the rate of R0 resection was comparable between the compared groups (89.7% and 91.7% in the macroscopic and microscopic subgroups, respectively). The proportion of patients alive at the last reported follow-up was identical in both subgroups (85.7%).

#### 3.1.3. Immunohistochemistry and Tumor Markers

As shown in Table 4, carcinoembryonic antigen (CEA) values were reported in 21 cases and carbohydrate antigen 19-9 (CA19-9) values in 17 cases. Increased CEA levels were reported in a number of patients, with levels reported from normal to 125.4 ng/mL. CA19-9 levels also showed great variability, ranging from normal values to 607.7 U/mL. Among the limited number of reported cases, CEA and CA19-9 levels were variably elevated and were normal or only mildly increased in some patients despite biliary involvement.

Immunohistochemical evaluation was available for 31 of 38 cases. The most common immunophenotype was CK7-negative and CK20-positive, observed in the majority of reported cases. More recent studies increasingly incorporated additional colorectal lineage markers, including CDX2 and SATB2, which were consistently positive when assessed. Nevertheless, atypical immunophenotypes, including CK7-positive/CK20-negative and CK7-negative/CK20-negative profiles, were reported in a small number of cases. Therefore, a CK7-negative/CK20-positive phenotype, particularly when supported by CDX2 and SATB2 expression together with the patient’s clinical history and histopathological findings, favors a colorectal origin over primary CCA rather than establishing the diagnosis in isolation.

#### 3.1.4. Long-Term Outcomes

**Figure 3:**
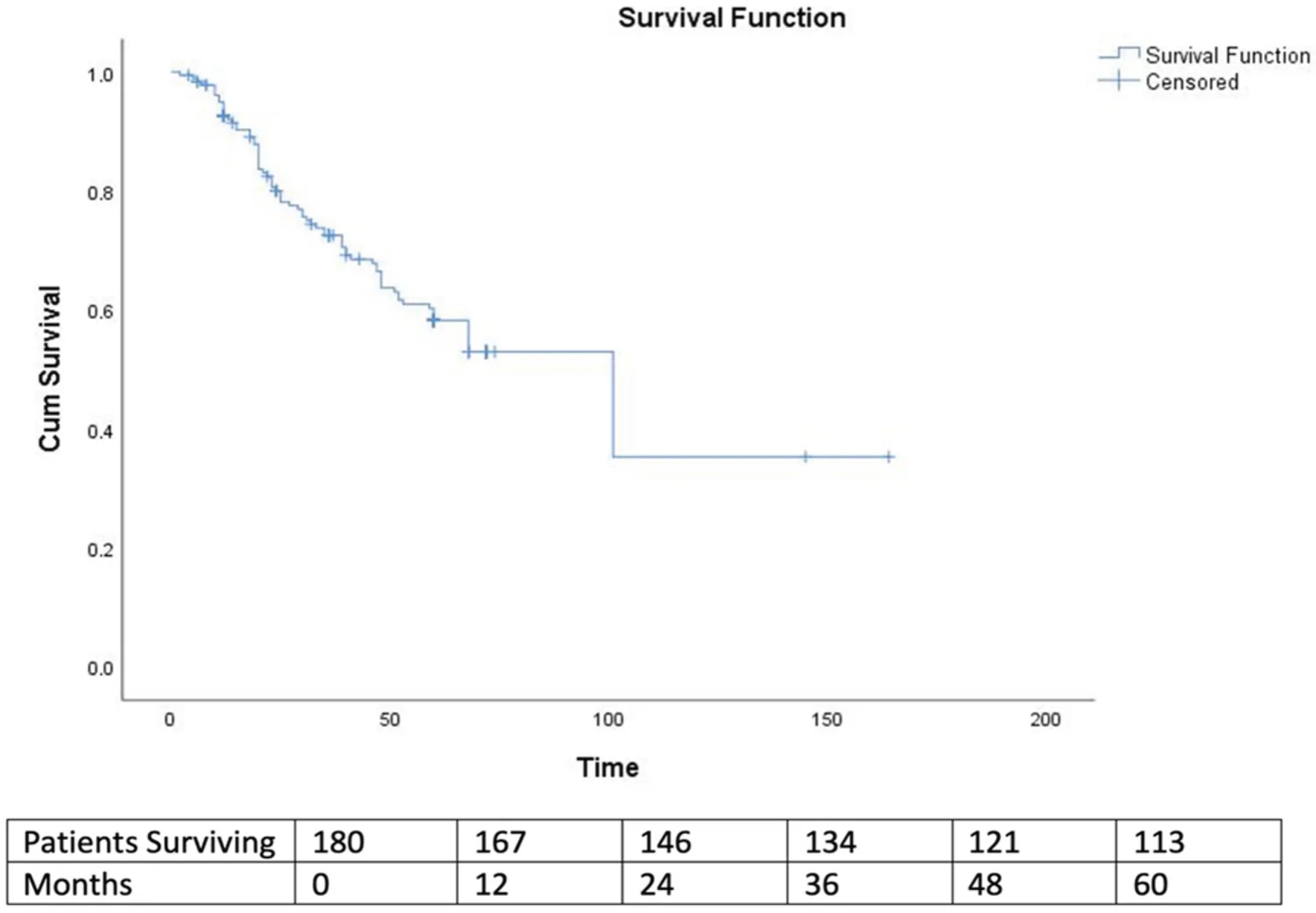
Kaplan–Meier curve of overall survival in patients with biliary colorectal liver metastases.

After a median follow-up of 52.5 months (range 2–164 months), 60.5% of patients were alive. Median survival for the entire cohort was 101 months (95% Confidence Interval: 49–153 months). Corresponding 3-year and 5-year survival rates were 74.4% and 62.8%, respectively (Figure 3). No statistically significant difference in terms of overall survival was demonstrable between the microscopic and macroscopic biliary invasion subgroups.

#### 3.1.5. Quality Assessment of Case Reports

Risk of bias was assessed with the Joanna Briggs Institute (JBI) Critical Appraisal Checklist for Case Reports. Overall, the methodological quality of the included studies was moderate, mainly due to sufficient reporting of patient demographics, diagnostic evaluation, clinical presentation, therapeutic interventions, and outcomes. However, most studies did not report detailed information on adverse events, complications, or long-term follow-up, which influenced the overall quality assessment. A number of studies were also rated high or unclear risk of bias due to incomplete follow-up data, vague description of timelines, or poor reporting of post-intervention outcomes.

#### 3.1.6. Quality Assessment of Case Series

Risk of bias for case series studies was assessed using the Joanna Briggs Institute (JBI) Critical Appraisal Checklist for Case Series (Supplementary Materials S2 and S3). Overall, most studies were considered to have moderate methodological quality. The included studies generally demonstrated adequate diagnostic confirmation methods and clear reporting of demographic and clinical characteristics. However, several studies lacked clearly defined inclusion criteria, consecutive patient inclusion, center demographic information, and comprehensive statistical analysis. In addition, outcome reporting and follow-up data were incomplete in some studies, contributing to an increased risk of bias. Three studies [8,39,46] were judged to have a higher risk of bias due to multiple unclear or missing methodological domains.

## 4. Discussion

This systematic review shows that ibCRLM are a rare but distinct clinicopathological subtype of colorectal liver metastases with different radiological, pathological, and surgical characteristics. Most lesions were metachronous and solitary, while microscopic biliary invasion was more frequently associated with synchronous and multifocal disease. Intrabiliary CRLM was often similar to iCCA, and they were especially difficult to diagnose preoperatively. However, characteristic imaging findings combined with immunohistochemical profiling can aid in diagnostic differentiation. Surgical resection remained the mainstay of treatment, with major hepatectomy being the most common procedure. Although technically demanding, selected patients undergoing curative-intent resection appeared to achieve encouraging long-term outcomes. However, these findings should be interpreted in light of the predominantly retrospective nature of the available evidence and the absence of comparative studies.

The immunohistochemical findings in the included case reports underscore the importance of pathological assessment for the diagnosis of ibCRLM. The radiological features were frequently indistinguishable from intrahepatic or extrahepatic CCA, and many cases could potentially be misdiagnosed. In this context, the characteristic CK7−/CK20+ immunophenotype, often supplemented by CDX2 and SATB2 positivity, represents a characteristic immunohistochemical profile supporting colorectal origin. These markers should be regarded as lineage markers that aid pathological differentiation from primary cholangiocarcinoma rather than as markers explaining the biological mechanism of intrabiliary extension. Their principal role is to confirm the colorectal origin of the lesion, particularly in patients presenting many years after treatment of the primary tumor, where metastatic disease may not be immediately suspected.

These findings are clinically relevant beyond diagnosis. Accurate distinction between ibCRLM and primary cholangiocarcinoma has direct implications for multidisciplinary decision-making, including selection of systemic therapies, prognostic counseling, and postoperative surveillance strategies. The increasing use of colorectal lineage markers such as CDX2 and SATB2 in recent publications also points to a trend toward a more accurate pathological characterization of these rare lesions. Thus, immunohistochemical profiling should be considered an integral component of the pathological evaluation of biliary lesions in patients with a present or previous history of colorectal cancer, facilitating confirmation of colorectal origin and differentiation from primary biliary malignancies.

To date, only one systematic review has been performed to evaluate ibCRLM, published by [54], which underscores the need for an updated synthesis of the literature. While their review mainly provided a descriptive review of 40 studies on radiological findings, pathology, and surgical management, our study provides a more detailed and methodologically rigorous analysis with an extended inclusion of the literature, standardized definitions, extraction of individual patient-level data, formal statistical analysis, and subgroup comparison of microscopic and macroscopic biliary invasion patterns. Unlike the prior review, which noted increased survival for patients with macroscopic biliary invasion, our analysis revealed similar survival rates across invasion subtypes, while also characterizing differences in tumor presentation and surgical management. Therefore, our study provides a more updated and detailed assessment of the clinicopathological features and outcome of this rare disease entity.

The present study has several strengths. To our knowledge, this is the largest systematic review of intrabiliary colorectal liver metastases (ibCRLMs) published to date. Despite the rarity of this disease, the use of individual patient-level data enabled a detailed characterization of tumor presentation, biliary invasion patterns, operative management, and survival outcomes. Nevertheless, several limitations should be acknowledged. The predominance of case reports and small retrospective case series introduces an inherent risk of publication and selection bias, as unusual clinical presentations and favorable surgical outcomes are more likely to be reported. In addition, many patients originated from specialized hepatobiliary centers, introducing potential referral bias that may limit the generalizability of our findings. Consequently, the results should be interpreted with caution and may not be representative of the broader population of patients with ibCRLMs. Furthermore, substantial heterogeneity in patient selection, operative strategies, definitions of clinicopathological variables, and outcome reporting precluded quantitative meta-analysis and limited the ability to perform formal sensitivity analyses. Moreover, several clinically relevant variables—including molecular tumor characteristics, recurrence patterns, perioperative systemic therapy, surgical margin status, and long-term follow-up—were inconsistently reported across studies, limiting the completeness of the descriptive analyses. Although duplicate publications were carefully assessed during study selection, the possibility of overlap between isolated case reports and institutional case series cannot be completely excluded. Finally, the absence of a comparator group of patients with conventional CRLM precludes definitive conclusions regarding the biological behavior or prognostic significance of intrabiliary tumor growth. Therefore, the findings of this review should be considered hypothesis-generating and interpreted within the context of the currently available low-level evidence.

The findings of this review have several practical implications for clinicians. Preoperatively, ibCRLM should be considered in patients with a history of colorectal cancer presenting with a liver lesion associated with segmental biliary dilatation, intraductal extension, or imaging features suggestive of iCCA. Recognition of these radiological features may facilitate appropriate multidisciplinary discussion and operative planning. Definitive differentiation from primary cholangiocarcinoma, however, relies on pathological assessment, with immunohistochemical markers such as CK7, CK20, CDX2, and SATB2 serving as lineage markers that support a colorectal origin rather than preoperative diagnostic tools. From a surgical perspective, careful preoperative assessment of the longitudinal extent of biliary involvement is essential to guide the need for bile duct resection or biliary reconstruction in order to achieve negative surgical margins. Conversely, pancreatoduodenectomy should be regarded as an exceptional procedure reserved for highly selected patients, as the currently available evidence is limited to only a small number of reported cases. Future collaborative multicenter registries with standardized reporting of radiological, pathological, surgical, and oncologic outcomes are needed to further clarify the biological behavior and optimal management of this rare disease entity.

**Figure 4:**
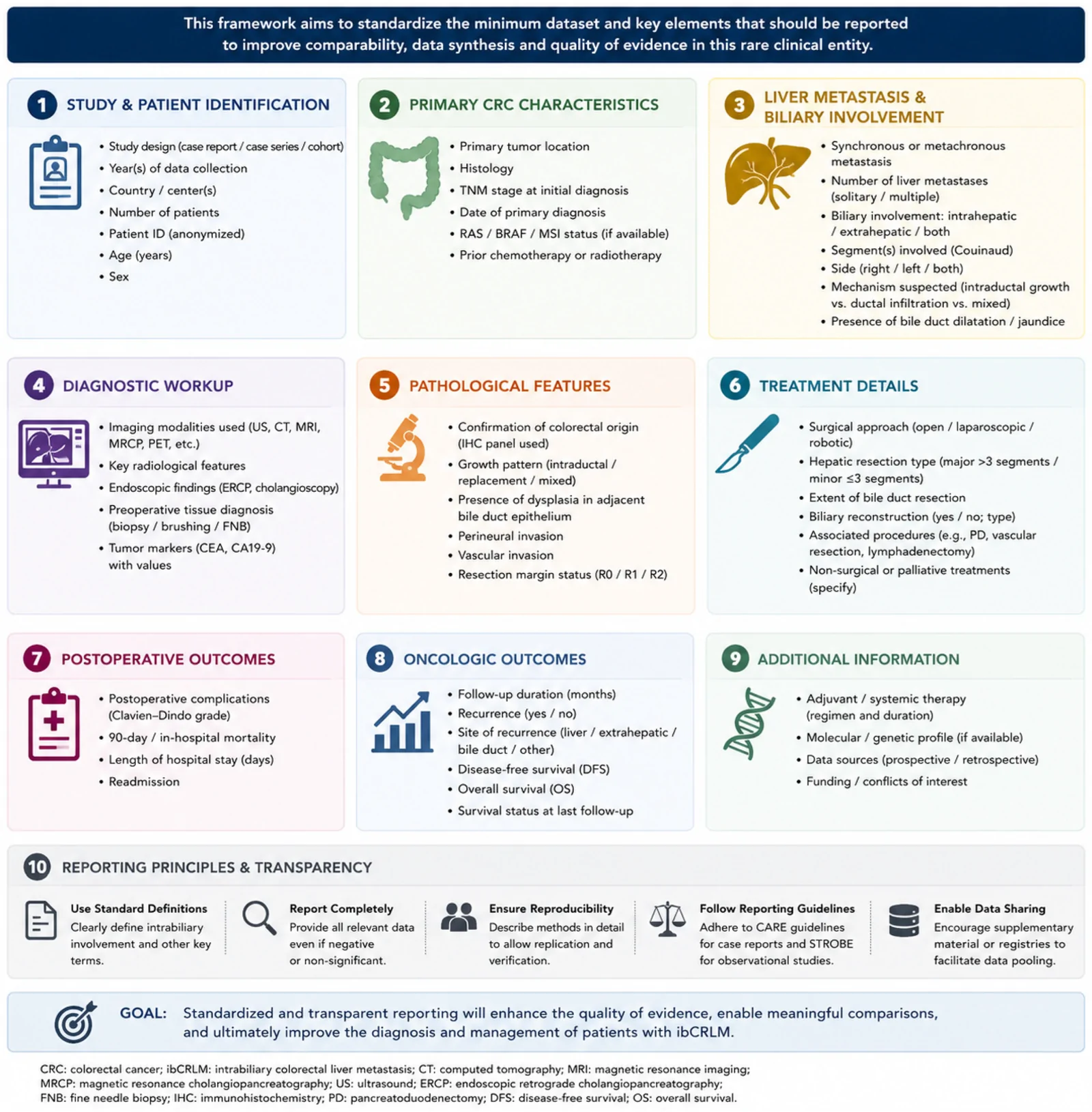
Proposed framework for standardized reporting of patients with ibCRLM. The framework summarizes the minimum dataset that should be reported in future studies, including patient demographics, primary tumor characteristics, radiological and pathological findings, treatment details, postoperative outcomes, oncological outcomes, and reporting principles to improve consistency and facilitate future evidence synthesis.

Future research should focus on the development of multicenter collaborative registries and standardized reporting frameworks for ibCRLM. There is currently significant heterogeneity in the definition of biliary invasion, radiologic interpretation, pathological classification, surgical techniques, and the reporting of oncological outcomes. Standardized reporting criteria for imaging findings, immunohistochemical profiling, surgical margins, recurrence patterns, molecular characteristics, and long-term survival outcomes would improve comparability between studies and allow pooled analyses in the future. Moreover, future studies evaluating the role of molecular profiling, novel systemic therapies, and minimally invasive hepatobiliary approaches may further shed light on the biology and optimal management of this rare clinical entity [Figure 4].

## 5. Conclusions

In conclusion, our analysis suggests that ibCRLM represents a distinct clinicopathological presentation of CRLM with characteristic radiological and pathological features. Their radiological resemblance to cholangiocarcinoma frequently makes preoperative diagnosis challenging, while immunohistochemical profiling plays a key role in confirming their colorectal origin. Accurate diagnosis and multidisciplinary evaluation are essential to guide appropriate surgical management. The available evidence suggests that selected patients with ibCRLM may achieve favorable long-term outcomes following complete surgical resection; however, current evidence remains insufficient to determine whether intrabiliary growth itself confers a biological or prognostic advantage over conventional colorectal liver metastases.

## Figures and Tables

**Table 1:** Baseline patient and tumor characteristics.

	Number of Patients Reported On	Number of Cases n, (%)
Mean age ± standard deviation	177	62.4 ± 10.9 years
Sex M/F	166	127 (76.5)/39 (23.5)
Case reports	38	38
Case series	10	190
**Presentation**		
*Solitary*	115	68 (59.1)
*Multinodular*	115	47 (40.9)
*Synchronous*	173	50 (28.9)
*Metachronous*	173	123 (71.1)
*Interval for presentation (range)*	116	30 (4–180) months *
**Primary tumor location**		
*Colon*	120	89 (74.2)
*Rectum*	120	31 (25.8)
**Staging of primary tumor**		
*Stage I*	53	4 (7.5)
*Stage II*	53	15 (28.3)
*Stage III*	53	24 (45.3)
*Stage IV*	53	10 (18.9)
**Tumor characteristics**		
*Microscopic biliary involvement*	205	120 (58.5)
*Macroscopic biliary involvement*	205	85 (41.5)
Size of involved tumors	158	3.2 ± 1.9 cm

* Values are presented as median (range). Abbreviations: M: Male; F: Female.

**Table 2:** Treatment-related characteristics from the entire patient cohort.

	Total Number of Patients Reported On	Number of Cases n, (%)
**Major hepatectomy**	93	43 (46.2)
**Minor hepatectomy**	93	36 (38.7)
**Pancreaticoduodenectomy**	93	4 (4.3)
**Palliative treatment**	93	10 (10.8)
**Biliary reconstruction**	93	27 (34.2)
**R0 resection**	120	54 (45)
**Patient status at last follow-up**		
*Alive*	180	109 (60.5)
*Deceased*	180	71 (39.5)

**Table 3:** Comparison between tumors demonstrating macroscopic versus microscopic biliary spread. * Categorical variables were compared using Fisher’s exact test, continuous variables using the Mann–Whitney U test, and overall survival using the log-rank test. Statistical significance was defined as *p* < 0.05.

	Macroscopic n, (%)	Microscopic n, (%)	*p*-Value
**Synchronous**	5/67 (7.5%)	20/55 (36.4%)	<0.001
**Solitary lesion**	34/47 (72.3%)	25/55 (45.5%)	0.008
**Size (cm)**	4 ± 1.5	3.7 ± 2.5	0.03
**Major hepatectomy**	19/39 (48.7%)	6/11 (54.5%)	0.8
**Minor hepatectomy**	5/39 (12.8%)	5/11 (45.5%)	0.03
**Biliary reconstruction**	4/39 (10.2%)	1/11 (9%)	0.9
**Palliative**	3/39 (7.7%)	0	0.8
**R0 resection rates**	26/29 (89.7%)	44/48 (91.7%)	1
**Survival at the end of follow-up**	12/14 (85.7%)	6/7 (85.7%)	0.3 *

**Table 4:** Tumor markers and immunohistochemical characteristics.

Author/Year	CEA/CA19-9	CK Profile
Sano et al., 2000 [42]	NR	NR
Wenzel et al., 2003 [51]	4.5/ND	CK20+, CK7−
Tomizawa et al., 2003 [48]	22/500	NR
Colagrande et al., 2004 [13]	36.6/120.6	CK20+, CK7−
Uehara et al., 2004 [50]	4.4/32	CK20+, CK7−
Takamatsu et al., 2004 [4]	NR	CK7−, CK20+
Tokai et al., 2006 [47]	NR	CK7−, CK20+
Lefevre et al., 2007 [29]	NR	CK7−, CK20+
Fraguela et al., 2007 [18]	5.8/83	CK7−, CK20+
Hiramatsu et al., 2007 [21]	NR	NR
Guettier et al., 2007 [20]	NR	CK7+, CK20−
Morelli et al., 2007 [31]	NR	CK7−, CK20+
Kayashima et al., 2008 [25]	125.4/107.9	CK7−, CK20+
Seshadri et al., 2009 [44]	Elevated/NR	CK7−, CK20+
Ghittoni et al., 2010 [19]	NR/241	NR
Kobayashi et al., 2011 [27]	6.2/NR	CDX2+, CK20+, CK7−
Nanashima et al., 2011 [34]	5.2/Normal	CK7−, CK20+
Nakamura et al., 2013 [33]	NR/607.71	CK7+, CK20−
Coppola et al., 2014 [15]	NR	CK20+, CDX2+, CK7−
Kawakatsu et al., 2015 [23]	NR	CK7−, CK20+
Yamao et al., 2015 [53]	NR	CK7+, CK20−
Dong et al., 2016 [16]	NR/Elevated	CK7−, CK20+
Kim et al., 2016 [26]	15.06/310.61	NR
Kon et al., 2016 [28]	6.8/40.3	CK7−, CK20−
Lopez Gordo et al., 2016 [30]	NR	NR
Rodriguez-Pascual et al., 2016 [41]	NR	NR
Conci et al., 2016 [14]	5.3/55	CDX2+, CK7−
Kawashima et al., 2017 [24]	5.2/ND	CK20−, CK7−
Anderloni et al., 2018 [11]	Normal/NR	NR
Hiroyoshi et al., 2018 [22]	8.7/NR	NR
Onishi et al., 2018 [37]	NR	CK20+, CDX2+, CK7−
Traeger et al., 2018 [49]	NR	CK20+, CK7−
Sasaki et al., 2019 [43]	21.4/174.5	CK7−, CK20+, CDX2+
Nakagawa et al., 2020 [32]	NR	CK7−, CK20+
Ofuchi et al., 2020 [36]	NR	CK7−, CK20+, CDX2+, SATB2+
Casas-Sicilia et al., 2021 [12]	50.3/2.48	CK7−, CK20+, CDX2+, SATB2+
Pereira da Silva et al., 2021 [38]	14.9/47.5	NR
O’Leary et al., 2023 [35]	2.7/23	CK20+, CDX2+, CK7−

Data availability: CEA values were reported in 21/38 cases (55.3%), CA19-9 values in 17/38 cases (44.7%), and immunohistochemical findings in 31/38 cases (81.6%). NR, not reported; ND, not determined.

## Data Availability

The data underlying this article derive from those included in the relevant published articles.

## Supplementary Materials

The following supporting information can be downloaded at: https://www.mdpi.com/article/10.3390/jpm16080436/s1, Supplementary Material S1: Preferred Reporting Items for Systematic Reviews and Meta-Analyses (PRISMA 2020) guidelines; Supplementary Materials S2 and S3: Risk of bias for case series studies was assessed using the Joanna Briggs Institute (JBI) Critical Appraisal Checklist for Case Series.

## Abbreviations

The following abbreviations are used in this manuscript:

CRC	Colorectal cancer
CRLM	Colorectal liver metastasis
iCCA	Intrahepatic cholangiocarcinoma
CT	Computed tomography
MR	Magnetic resonance
ibCRLM	Intra-biliary colorectal liver metastasis
MDT	Multidisciplinary team
PTCD	Percutaneous transhepatic biliary drainage
CEA	Carcinoembryonic antigen
CA 19-9	Carbohydrate antigen 19-9
