# Supplementary material for: Disease Characteristics and Management of Intrabiliary Colorectal Liver Metastasis: An Updated Systematic Review
Source: J Pers Med. 2026 Aug 20;16(8):436. doi: 10.3390/jpm16080436 (PMC13514391; doi:10.3390/jpm16080436)
Supplement: Supplementary file 1 [file jpm-16-00436-s001.zip › jpm-4396308-supplementary.pdf]

PubMed

(("colorectal liver metastases"[Title/Abstract] OR "colorectal liver metastasis"[Title/Abstract] OR CRLM[Title/Abstract] OR "liver metastases"[Title/Abstract] OR "hepatic metastases"[Title/Abstract]) AND (colorectal[Title/Abstract] OR colon[Title/Abstract] OR rectal[Title/Abstract] OR rectum[Title/Abstract]) AND (intrabiliary[Title/Abstract] OR "intra-biliary"[Title/Abstract] OR "biliary growth"[Title/Abstract] OR "intrabiliary growth"[Title/Abstract] OR "bile duct invasion"[Title/Abstract] OR "bile duct involvement"[Title/Abstract] OR "biliary invasion"[Title/Abstract] OR "biliary tree"[Title/Abstract] OR "intraductal growth"[Title/Abstract] OR "intraductal metastasis"[Title/Abstract]))

EMBASE

('colorectal liver metastasis'/exp OR 'colorectal liver metastases':ti,ab OR 'colorectal liver metastasis':ti,ab OR crlm:ti,ab OR 'liver metastases':ti,ab OR 'hepatic metastases':ti,ab) AND (colorectal:ti,ab OR colon:ti,ab OR rectal:ti,ab OR rectum:ti,ab) AND (intrabiliary:ti,ab OR 'intra-biliary':ti,ab OR 'intrabiliary growth':ti,ab OR 'biliary growth':ti,ab OR 'bile duct invasion':ti,ab OR 'bile duct involvement':ti,ab OR 'biliary invasion':ti,ab OR 'biliary tree':ti,ab OR 'intraductal growth':ti,ab OR 'intraductal metastasis':ti,ab)

CENTRAL

TS=(("colorectal liver metastases" OR "colorectal liver metastasis" OR CRLM OR "liver metastases" OR "hepatic metastases") AND (colorectal OR colon OR rectal OR rectum) AND (intrabiliary OR "intra-biliary" OR "intrabiliary growth" OR "biliary growth" OR "bile duct invasion" OR "bile duct involvement" OR "biliary invasion" OR "biliary tree" OR "intraductal growth" OR "intraductal metastasis"))

Web of Science

TS=(("colorectal liver metastases" OR "colorectal liver metastasis" OR CRLM OR "liver metastases" OR "hepatic metastases") AND (colorectal OR colon OR rectal OR rectum) AND (intrabiliary OR "intra-biliary" OR "intrabiliary growth" OR "biliary growth" OR "bile duct invasion" OR "bile duct involvement" OR "biliary invasion" OR "biliary tree" OR "intraductal growth" OR "intraductal metastasis"))

CINAHL

(("colorectal liver metastases" OR "colorectal liver metastasis" OR CRLM OR "liver metastases" OR "hepatic metastases") AND (colorectal OR colon OR rectal OR rectum) AND (intrabiliary OR "intra-biliary" OR "intrabiliary growth" OR "biliary growth" OR "bile duct invasion" OR "bile duct involvement" OR "biliary invasion" OR "biliary tree" OR "intraductal growth" OR "intraductal metastasis"))

S2: JBI for case reports									
**Study**	**D1 Demographics**	**D2 History/Timeline**	**D3 Clinical Condition**	**D4 Diagnostic Tests**	**D5 Intervention**	**D6 Post-intervention Condition**	**D7 Adverse Events**	**D8 Takeaway/Lessons**	**Overall risk of bias**
Sano et al., 2000	Yes	Yes	Yes	Yes	Yes	Yes	No	Yes	Moderate
Wenzel et al., 2003	Yes	Yes	Yes	Yes	Yes	Yes	No	Yes	Moderate
Tomizawa et al., 2003	Yes	Yes	Yes	Yes	Yes	Yes	No	Yes	Moderate
Colagrande et al., 2004	Yes	Partial	Yes	Yes	Yes	No	No	Partial	High
Uehara et al., 2004	Yes	Yes	Yes	Yes	Yes	Yes	No	Yes	Moderate
Takamatsu et al., 2004	Yes	Yes	Yes	Yes	Yes	Partial	No	Yes	Moderate
Tokai et al., 2006	Yes	Yes	Yes	Yes	Yes	Yes	No	Yes	Moderate
Lefevre et al., 2007	Yes	Yes	Yes	Yes	Yes	Yes	No	Yes	Moderate
Fraguela et al., 2007	Yes	Yes	Yes	Yes	Yes	Yes	No	Yes	Moderate
Hiramatsu et al., 2007	Yes	Partial	Yes	Yes	Yes	Yes	No	Partial	Moderate
Guettier et al., 2007	Yes	No	Yes	Partial	Partial	No	No	Partial	High
Morelli et al., 2007	Yes	Partial	Yes	Yes	Yes	Yes	No	Yes	Moderate
Kayashima et al., 2008	Yes	Yes	Yes	Yes	Yes	Yes	No	Yes	Moderate
Seshadri et al., 2009	Yes	Yes	Yes	Yes	Yes	Yes	No	Yes	Moderate
Ghittoni et al., 2010	Yes	Yes	Yes	Yes	Yes	Yes	No	Yes	Moderate
Kobayashi et al., 2011	Yes	Yes	Yes	Yes	Yes	Yes	No	Yes	Moderate
Nanashima et al., 2011	Yes	Yes	Yes	Yes	Yes	No	No	Partial	High
Nakamura et al., 2013	Yes	Yes	Yes	Yes	Yes	Yes	No	Yes	Moderate
Coppola et al., 2014	Yes	Yes	Yes	Yes	Yes	Yes	No	Yes	Moderate
Kawakatsu et al., 2015	Yes	Yes	Yes	Yes	Yes	Yes	No	Yes	Moderate
Yamao et al., 2015	Yes	Yes	Yes	Yes	Yes	No	No	Partial	High
Dong et al., 2016	Yes	Yes	Yes	Yes	Yes	Partial	No	Partial	High
Kim et al., 2016	Yes	Yes	Yes	Yes	Yes	No	No	Partial	High
Kon et al., 2016	Yes	Yes	Yes	Yes	Yes	Yes	No	Yes	Moderate
Lopez Gordo et al., 2016	Yes	Partial	Yes	Partial	Yes	Yes	No	Partial	Moderate
Rodriguez Pascual et al., 2016	Yes	Partial	Yes	Yes	Partial	Yes	No	Partial	High
Conci et al., 2016	Yes	Yes	Yes	Yes	Yes	No	No	Partial	High
Kawashima et al., 2017	Yes	Yes	Yes	Yes	Yes	No	No	Partial	High
Traeger et al., 2018	Yes	Partial	Yes	Yes	Partial	No	No	Partial	High
Anderloni et al., 2018	Yes	Yes	Yes	Yes	Yes	Yes	No	Yes	Moderate
Onishi et al., 2018	Yes	Partial	Yes	Yes	Yes	Yes	No	Partial	Moderate
Hiroyoshi et al., 2018	Yes	Yes	Yes	Yes	Yes	Yes	No	Yes	Moderate
Sasaki et al., 2019	Yes	Yes	Yes	Yes	Yes	Yes	No	Yes	Moderate
Ofuchi et al., 2020	Yes	Yes	Yes	Yes	Yes	No	No	Partial	High
Nakagawa et al., 2020	Yes	Yes	Yes	Yes	Yes	Yes	No	Yes	Moderate
O’Leary et al., 2021	Yes	Yes	Yes	Yes	Yes	Yes	No	Yes	Moderate
Silva et al., 2021	Yes	Partial	Yes	Yes	Partial	No	No	Partial	High

Casas-Sicilia et al., 2021	Yes	Yes	Yes	Yes	Yes	Yes	No	Yes	Moderate

S3: JBI for case series

Study	D1 Inclusion Criteria	D2 Standard Measurement	D3 Valid Condition Identification	D4 Consecutive Inclusion	D5 Complete Inclusion	D6 Demographics Reported	D7 Clinical Information Reported	D8 Outcomes/Follow-up Reported	D9 Site Demographics Reported	D10 Statistical Analysis	Overall risk of bias
Riopel et al., 1997	No	Yes	Yes	Unclear	Unclear	Yes	Yes	Partial	No	No	High
Okano et al., 1999	Partial	Yes	Yes	Unclear	Unclear	Yes	Yes	Yes	No	Partial	Moderate
Povoski et al., 1999	No	Yes	Yes	Unclear	Unclear	Yes	Yes	Yes	No	No	High
Kubo et al., 2002	Partial	Yes	Yes	Unclear	Unclear	Yes	Yes	Partial	No	Partial	Moderate
Sugiura et al., 2006	No	Yes	Yes	Unclear	Unclear	Yes	Yes	Yes	No	No	Moderate
Estrella et al., 2013	Partial	Yes	Yes	Unclear	Partial	Yes	Yes	Yes	No	Partial	Moderate
Tirapu et al., 2014	No	Yes	Yes	Unclear	Yes	Yes	Yes	Partial	No	No	High
Wiggers et al., 2016	Partial	Yes	Yes	Unclear	Partial	Yes	Yes	Yes	No	Partial	Moderate
Reijonen et al., 2019	Partial	Yes	Yes	Unclear	Partial	Yes	Yes	Yes	No	Partial	Moderate
Zeng et al., 2021	Partial	Yes	Yes	Unclear	Yes	Yes	Yes	Partial	No	No	Moderate
